# Brazilian red propolis as a potential antimicrobial additive in orthodontic primers

**DOI:** 10.1590/0103-644020256036

**Published:** 2025-04-07

**Authors:** João Marcos Spessoto Pingueiro, Murilo Fernando Neuppmann Feres, Carla Cristina Macedo Vaz-Monteiro, Helen Karine Vieira-Silva, Gustavo Quilles Vargas, Larissa Matias Malavazi, Pedro Luiz Rosalen, Jamil Awad Shibli, Marina Guimarães Roscoe, Bruno Bueno-Silva

**Affiliations:** 1 Dental Research Division, School of Dentistry, Guarulhos University, Guarulhos, São Paulo, Brazil.; 2 Department of Orthodontics and Pediatric Dentistry, Faculty of Dentistry, University of São Paulo, São Paulo, São Paulo, Brazil; 3 Department of Bioscience, Piracicaba Dental School, University of Campinas, Piracicaba, São Paulo, Brazil; 4 Department of Food and Medicine, Federal University of Alfenas, Alfenas, Minas Gerais, Brazil; 5 Faculty of Dentistry, Oral and Craniofacial Sciences, King’s College London, London, UK

**Keywords:** propolis, dental adhesive, microbiology, antimicrobial, shear bond strength

## Abstract

This study evaluated the effect of adding ethanolic extract of red propolis (EERP) on antimicrobial activity, polymerization kinetics (PK), degree of conversion (DC), and shear bond strength (SBS) of the orthodontic adhesive system. Transbond XT Primer was the control group (G1). The experimental adhesives contained 3.25 mg/mL (G2) and 6.50 mg/mL (G3) EERP. Minimum inhibitory concentration and biofilm assay against *Streptococcus mutans*, and bacterial and exopolysaccharide (EPS) evaluation by confocal laser scanning microscope were performed. PK and DC were evaluated by Fourier Transform Infrared Spectroscopy. The bracket-tooth SBS was evaluated by a universal testing machine. Both antimicrobial and SBS results were analyzed by ANOVA and Tukey's *post-hoc* test (p<0.05). PK and DC data were submitted to ANOVA and Bonferroni *post-hoc* test. G3 presented better antimicrobial activity, inhibiting more than 95% of planktonic *S. Mutans*, 25% of biofilm dry-weight, and 50% of EPS production (p<0.05). PK was different among the groups. DC after 40 seconds of photoactivation ranged from 68.62% for G1 to 76.85% for G2 (p<0.05). SBS data was not presented as statistically significant (p>0.05). Transbond XT Primer modified with 6.5 mg/mL EERP demonstrated antimicrobial activity with no reduction of the bracket-tooth SBS. Propolis-modification did not influence the physic-chemical properties of the orthodontic adhesive.

## Resumo

Este estudo avaliou o efeito da adição de extrato etanólico de própolis vermelha (EERP) na atividade antimicrobiana, cinética de polimerização (PK), grau de conversão (DC) e resistência ao cisalhamento (SBS) do sistema adesivo ortodôntico. O grupo controle foi o Transbond XT Primer (G1). Os adesivos experimentais continham 3,25 mg/mL (G2) e 6,50 mg/mL (G3) de EERP. Foi efetuada a concentração inibitória mínima e o ensaio de biofilme contra *Streptococcus mutans*, bem como a avaliação bacteriana e do exopolissacarídeo (EPS) através do microscópio confocal de varredura a laser. A PK e a DC foram avaliadas por espectroscopia de infravermelhos com transformada de Fourier. O SBS do braquete ao dente foi avaliado por uma máquina de testes universal. Os resultados antimicrobianos e de SBS foram analisados por ANOVA e teste *post-hoc* de Tukey (p<0,05). Os dados de PK e DC foram submetidos à ANOVA e ao teste *post-hoc* de Bonferroni. O G3 apresentou melhor atividade antimicrobiana, inibindo mais de 95% do *S. Mutans* planctônico, 25% do peso seco do biofilme e 50% da produção de EPS (p<0,05). As PK foram diferentes entre os grupos. A DC após 40 segundos de fotoativação variou de 68,62% para G1 a 76,85% para G2 (p<0,05). Os dados da EBE não apresentaram significância estatística (p>0,05). O Transbond XT Primer modificado com 6,5 mg/mL de EERP demonstrou atividade antimicrobiana sem redução da SBS do braquete ao dente. A modificação com própolis não influenciou as propriedades físico-químicas do adesivo ortodôntico.

**Figure:**
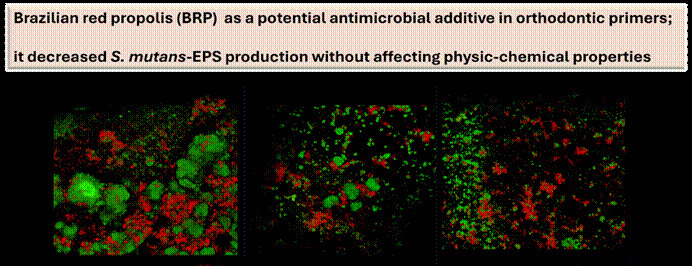

## Introduction

Dental orthodontic fixed appliances might result in biofilm accumulation with consequent white spot lesions, due to the increased number of retention sites, *S. Mutans* adhesion, inadequate oral hygiene, and pH decrease ^1^ ^,^ ^2^. It has been reported that white spot lesions (first sign of caries) prevalence may increase from 46% to 59% after one year of orthodontic treatment ^3^. Hence, patients undergoing orthodontic full-fixed appliance treatment might require not only regular oral hygiene instruction, but also the application of fluoride varnish, chlorhexidine, and dietary modification ^4^. These procedures require optimal patient adhesion and collaboration, and orthodontic patients are mostly teenagers who present limited dexterity and motivation ^5^.

This scenario highlights the importance of developing materials intended to prevent demineralization, stimulate remineralization, or control patient- and biofilm-related factors, with less dependence on patient compliance. In this sense, several antibacterial orthodontic adhesive systems have been developed ^6^, including the Transbond adhesive primmer that has triphenyl antimony in its composition. This substance presented antimicrobial activity against several gram-positive bacteria and also against *S. Mutans* biofilm ^7^ ^,^ ^8^ however, literature still seeks strategies to improve the antimicrobial properties of different dental materials ^9^ ^,^ ^10^. For instance, the addition of fluoride and chlorhexidine has already been tested; however, these components present a short-term release of antimicrobial agents, in addition to negatively interfering with mechanical properties ^11^ ^,^ ^12^.

Natural products might also be considered as potential agents, due to their anti-cariogenic activity and non-toxicity. Propolis is a non-toxic resinous substance that can be collected from plants by bees and presents potential antimicrobial, anti-caries, and anti-inflammatory properties. In Brazil, a specific type of propolis from Alagoas State is called Brazilian red propolis and is classified as type 13 ^13^ ^,^ ^14^. The Brazilian red propolis has already demonstrated antimicrobial properties against bacteria related to caries and periodontal disease, as its anti-*S. Mutans* biofilm and anti-caries effect were similar as observed to fluoride, according to an animal model ^15^ ^,^ ^16^ ^,^ ^17^. Based on the above, the present study hypothesizes that Brazilian red propolis enhances the antimicrobial property of an orthodontic primer; since the use of Brazilian red propolis as an additive to orthodontic materials was still not evaluated thus far. Regarding potential mechanisms of action of Brazilian red propolis, the literature indicates that in the planktonic state of *S. Mutans*, Brazilian red propolis presented bactericidal activity. However, in biofilms, it appears that Brazilian red propolis exerts its effect through the inhibition of virulence factors, including glycosyltransferases and exopolysaccharide production, without affecting bacterial viability but decreasing biofilm formation ^13^ ^,^ ^14^ ^,^ ^15^.

Therefore, this *in vitro* study was aimed at evaluating the effect of ethanolic extract of red propolis (EERP) addition on antimicrobial activity, polymerization kinetics, degree of conversion (DC), and shear bond strength (SBS) of a conventional orthodontic adhesive system.

## Materials and methods

### Preparation of EERP and the experimental groups

Samples of red propolis type 13 were geo-referenced and collected by scraping multiple *Apis mellifera* beehives from Marechal Deodoro, a city located in Maceió surroundings, at Alagoas State (Brazil). Governmental authorization to research Brazilian red propolis was obtained by SISGEN number A305815. The propolis extracts were prepared as described elsewhere. The crude extract was prepared by mixing 25 g of crude red propolis in 200 mL of 80% (v/v) ethanol, macerating it at 45 °C for 30 minutes. After this procedure, the mixture was centrifuged, and filtered and the supernatant was evaporated under pressure to remove the solvent. The extract remaining in the flask was the EERP ^17^. Afterwards, it was prepared in 10 mg/mL and 20 mg/mL to be incorporated into the orthodontic adhesive (Transbond XT primer, 3M ESPE, Saint Paul, Minnesota, USA). After incorporation, the EERP final concentration was 3.25 mg/mL and 6.5 mg/mL. The concentrations were selected based on the findings of previous studies on Brazilian red propolis compounds (neovestitol and vestitol) and their effects on *S. Mutans* biofilm ^15^. The compounds demonstrated noteworthy outcomes at 800 µg/mL, and in this study, approximately 4x and 8x the concentrations were employed.

A conventional orthodontic bonding adhesive (Transbond XT; 3M Unitek, Monrovia, California, USA) was used and as a control group, the Transbond XT primer was used (G1). This material is essentially composed of bisphenol A diglycidyl ether dimethacrylate (bis-GMA) (34%-55%), and triethylene glycol dimethacrylate (TEGDMA) (45%-55%). As for the other groups, 3.25 mg (G2) and 6.5 mg (G3) of EERP were incorporated into 1 ml of Transbond XT primer in sealed bottles, after a 12-hour stirring at room temperature. The final concentration of ethanol when added to media containing bacteria was approximately 3%. These concentrations were chosen by previous results with *S. Mutans* biofilm.

After this period, specimens of each of the three groups were used in the antimicrobial test, as described in the following section. After the experiment, specimens were stored under refrigeration (8 ^ο^C - 10 ^ο^C) and the antimicrobial tests were repeated after three months of storage.

The experimental groups were dispensed until they filled a Teflon matrix. Thereafter, a polyester strip was positioned over the dispensed adhesive; and on top of it, a glass slide was positioned for excess removal, as well as prevention of the contact of the fluid adhesive with the atmospheric oxygen during the photopolymerization. The photoactivation was performed for 40 seconds using a light-emitting diode (LED RADII; SDI, Bayswater, Australia) with an irradiance of 1200 mW/cm², calibrated regularly with the radiometer (Curing Radiometer; Demetron Corp., Danbury, Connecticut, USA), with the tip positioned perpendicular to the surface of the specimen.

### Antimicrobial activity test

*S. Mutans* UA159 was reactivated from stock cultures in Brain Heart Infusion Media (BHI) at 37 °C, 5% CO_2,_ for 24 hours, and grown on BHI agar plates. After microbial growth, 5-10 individual colonies were inoculated in BHI overnight. Afterwards, *S. Mutans* growth curve were performed until log phase (1-2 x 10_8_ cfu/mL) ^13^ ^,^ ^18^.

A volume of 100 μL of log phase microbial suspension was inoculated into 100 mL of BHI to obtain 1-2 x 10_5_ cfu/mL inoculum. Immediately after homogenization, a volume of 200 μL of medium plus inoculum was added to each well of 96-well microplate with the experimental composites modified by different concentrations of propolis.

Microplates were incubated at 5% CO_2_, 37 °C, for 24 hours. After incubation, the microplates were shaken, and the experimental composites removed and were read through an ELISA reader. There were two controls: microorganism inoculated culture medium and culture medium (without inoculum), used as blank for reading. Microorganism inoculated culture was considered as 100% of bacteria growth, and results were presented as the percentage of *S. Mutans* inhibition. As mentioned before, this test was repeated after three months of specimens’ storage.

### Anti-*S. Mutans* biofilm effect

Upper incisors metallic orthodontic brackets, Light versions, Roth prescription, .022” (Morelli, Sorocaba, São Paulo, Brazil) were bonded by propolis modified and control adhesive to hydroxyapatite discs, used as a substrate for biofilm formation, as described in the following section. The photoactivation was performed for 40 seconds using a light-emitting diode (LED RADII; SDI, Bayswater, Australia) with an irradiance of 1200 mW/cm², calibrated regularly with the radiometer (Curing Radiometer; Demetron Corp., Danbury, Connecticut, USA), with the tip positioned perpendicular to the surface of the hydroxyapatite disc.

A *S. Mutans* growth curve was performed as described for the previous assay. A volume of 100 μL of the microbial suspension was inoculated into 100 mL of BHI medium plus 1% sucrose to obtain 1-2 x 10^5^ cfu/mL inoculum. Immediately after homogenization, a volume of 2.5 mL of medium plus the inoculum was added to each well of the 24-well microplate along with the hydroxyapatite disks ^19^.

The monospecies biofilms of *S. Mutans* UA 159 were formed on hydroxyapatite discs (diameter 12.3 mm, thickness of 1.43 mm; Clarkson Calcium Phosphates, Williamsport, Pennsylvania, USA) with a bracket placed on the disc using the three evaluated adhesive systems. These were inserted into microplates with wells containing Brain Heart Infusion (BHI, Difco) culture medium with 1% sucrose, and incubated at 37 °C, 5% CO^2^, for 116 hours (5 days) to form the biofilm. Culture media were daily changed. At the end of day 5, biofilms were sonicated, and diluted, and the resultant biomass (dry weight) and microbial counts (cfu/mL) were assessed ^17^.

### Confocal laser scanning microscope analysis for *S. Mutans* biofilm

The biofilms were also analyzed by a confocal laser scanning microscope (CLSM - Zeiss LSM 780-NLO, Carl Zeiss Microscopy GmbH, Jena, Germany) after two days (48 hours) of formation. The extracellular polysaccharide produced by *S. Mutans* was triggered by the daily inclusion of Alexa Fluor 647 dextran conjugate (Life Technologies, Carlsbad, CA, USA) in the culture media. In addition, thirty minutes previously to confocal imaging, the dye SYTO 9 (Life Technologies, Carls-bad, CA, USA) was added to the media to stain bacterial cells (absorbance/fluorescence emission maxima of 647/668 nm and 485/498 nm, respectively). The biofilm was preserved intact and examined using CLSM equipped with an EC Plan-Neofluar 20× water immersion objective lens. Each disk was examined at three random points, and a 3D image was created by obtaining serial images in depth (z stack - 4 µm intervals). The confocal image stacks were analyzed with COMSTAT, which generates biofilm biomass measurements for quantifying the bacterial and EPS portion, thus characterizing the 3D structure of biofilms ^15^ ^,^ ^19^.

### Polymerization kinetics and degree of conversion (DC)

To evaluate the polymerization behavior of the orthodontic adhesives, seven samples per group were tested. Polymerization kinetics and DC of the materials were evaluated using Fourier-transform infrared spectroscopy (FTIR) with a spectrometer (Bruker Tensor 27 FT-IR, Massachusetts, EUA) equipped with an attenuated total reflectance device, composed of a horizontal diamond crystal. Adhesive droplets (about 3 µL) of control (G1) and experimental groups (G2 and G3) were dispensed directly onto the diamond crystal and light-cured for 40 seconds using a light-emitting diode (LED RADII; SDI, Bayswater, Australia) with an irradiance of 1200 mW/cm², regularly checked with radiometer (Demetron, Danbury, USA). One scan was acquired every second during photoactivation. Analyses were performed in a light-controlled room with a temperature of 23 °C (± 2 °C).

The DC was calculated based on the intensity of the carbon-carbon double-bond stretching vibrations (peak height) at 1635 cm^−1^ and using the symmetric ring stretching at 1610 cm^−1^ from the polymerized and non-polymerized samples as an internal standard ^20^. Data were plotted and curve fitting was applied using logistic non-linear regression. In addition, the polymerization rate (Rp (s−1)) was calculated as the degree of conversion at time t subtracted from the degree of conversion at time t - 1.

### Bracket-tooth shear bond strength (SBS)

Thirty freshly extracted healthy bovine incisors were cleaned with periodontal curettes (Millenium Golgran, São Caetano do Sul, São Paulo, Brazil) and divided into three groups (n=10). According to national law, there is no need for further permission to collect the bovine incisors for research. Prophylaxis was performed using rubber cups (Microdont, São Paulo, São Paulo, Brazil) with pumice paste (SS White, Petrópolis, Rio de Janeiro, Brazil) for ten seconds. The teeth were posteriorly washed and dried for standardized times. The rubber cups were replaced every five prophylaxes, to ensure quality and standardization of this procedure.

Teeth surfaces were then etched with 37% phosphoric acid (Dentsply, York, Pennsylvania, USA) for 30 seconds. The etching was performed in the center of the buccal surface, in a standardized area corresponding to the size of the base of the bracket used. After etching, the teeth were washed for 60 seconds, and dried. A thin layer of the orthodontic adhesives (G1, G2, and G3) was applied to the etched surfaces and then air-dried. Afterward, Transbond XT composite (3M ESPE, Saint Paul, Minnesota, USA) was applied to the bracket's bases (Morelli, Sorocaba, São Paulo, Brazil). The orthodontic brackets were pressed to the center of the buccal face, followed by excess removal. The composite was then light cured for 20 seconds (Radii-Cal; SDI, Australia) with an irradiance of 1200 mW/cm², regularly checked with a radiometer (Demetron, Danbury, Pennsylvania, USA).

To assist the specimens’ preparation for the SBS evaluation (embedding and alignment of the specimen inside the PVC pipe), a device with vertical displacement (Odeme Dental Research, Luzerna, Santa Catarina, Brazil), which contains a system for fixing the tooth-bracket into a rectangular orthodontic wire, was used to guarantee perpendicularity of the specimen and evaluation of the adhesive interface. The specimens were then saved in distilled water at 37°C for 72 hours. After this period, they were submitted to the SBS test.

A universal testing machine (Shimadzu, Kyoto, Japan) was utilized for the mechanical test. The samples were positioned on a shear device with a knife-edged chisel (Odeme Dental Research, Luzerna, Santa Catarina, Brazil). A compressive load was applied at the enamel-bracket interface at a 0.5 mm/min-crosshead speed until the bracket debonding. The test outputs were in kilogram-force (kgf), and SBS values were converted into megapascals (MPa), which considered the bracket base area.

After the SBS test, the bracket bases were examined using a stereomicroscope at 4x magnification (Stemi 508; Zeiss, Göttingen, Germany). The adhesive remnant index (ARI) was used to quantify the amount of adhesive left on the enamel surfaces (“0”: no adhesive; “1”: less than half; “2”: more than half; and “3”: total enamel bonding site covered with adhesive).

### Statistical analysis

Differences in SBS, antimicrobial activity, dry weight, and CLSM of biofilms were analyzed with the one-way ANOVA, and Tukey's *post-hoc* test. The polymerization kinetics was graphically described and DC data were submitted to one-way ANOVA and Bonferroni test. Fisher's exact test was performed to analyze differences between groups about ARI scores. Statistical significance was set at 5%.

## Results

### Antimicrobial activity

**Figure 1:**
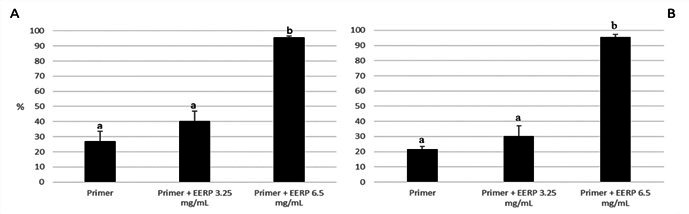
Antimicrobial activity of primer and primer modified by ethanolic extract of red propolis (EERP) at 3.25 and 6.5 mg/mL right after its production (A) and after three months of storage (B) (n = 6). Different letters mean statistical significance by Analysis of variance (ANOVA) followed by Tukey-test.

The addition of 6.5 mg/mL of EERP to the commercial adhesive (G3) inhibited more than 95% of planktonic *S. Mutans* growth. This percentage was statistically different (p < 0.05) in comparison with the remaining groups (G1 and G2). These results were observed immediately after the adhesive production (Figure 1A), as well as after three months of storage (Figure 1B).

### *S. Mutans* biofilm

**Figure 2:**
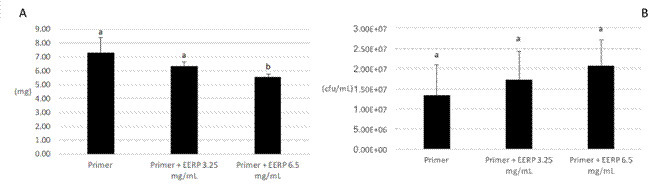
Dry-weight (A) and colony forming unit (B) of biofilms formed on hydroxyapatite discs with a bracket attached with primer, primer modified by EERP 3.25 mg/mL and primer modified by EERP 6.5 mg/mL (n = 6). Different letters mean statistical significance by ANOVA followed by Tukey-test.

The addition of 6.5 mg/mL of EERP to the commercial primer increased the antimicrobial activity. This group (G3) presented statistically lower dry-weight of biofilm formed on hydroxyapatite discs with brackets when compared to biofilm formed on discs plus brackets attached with conventional adhesive (G1) or modified by EERP 3.25 mg/mL (G2) (p < 0.05) (Figure 2A). However, there were no significant differences between all of the groups in relation to colony-forming unit (CFU) counting (p > 0.05) (Figure 2B).

**Figure 3:**
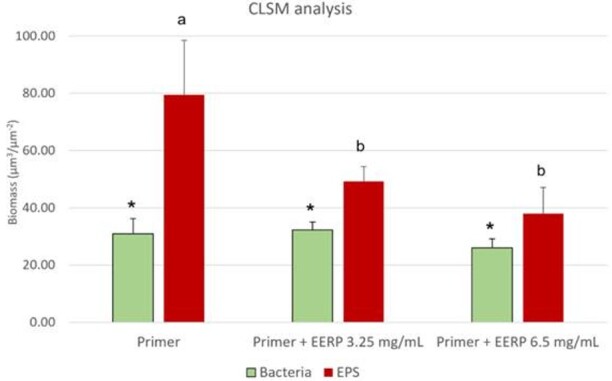
Quantitative analysis of bacterial and EPS biomass by COMSTAT software using confocal images of biofilms (n = 6). Statistical analysis was performed using ANOVA, with the Tukey test. * mean no statistical significance among groups of a bacterial portion of biofilms. Different letters mean statistical significance among groups of EPS portion of biofilms.

Both modified primers (G2 and G3) significantly reduced the biomass of EPS when compared to control commercial primer by approximately 40 and 53%, respectively (Figure 3). The bacterial portion of biofilms formed on the three groups did not have any statistical significance (Figure 3) what corroborates the CFU data (Figure 2B).

**Figure 4:**
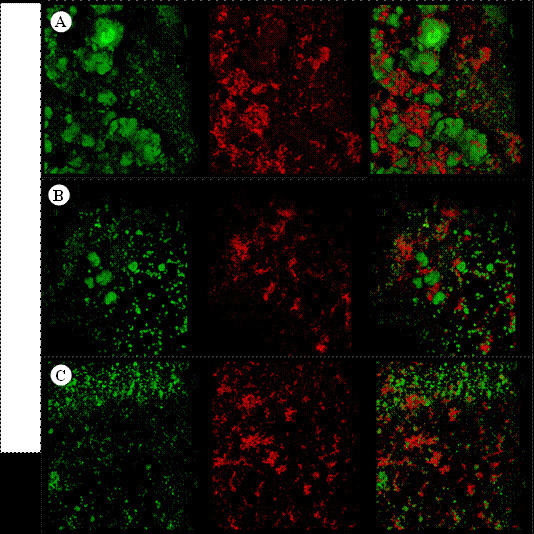
Representative rendered images of *S. Mutans* biofilm formed for 48 hours. The first column shows cells depicted in green (SYTO 9) representing the bacterial portion, the second column shows the EPS matrix, depicted in red (Alexa Fluor 647 dextran) and the third column shows the overlaid biofilms images (Bacteria +EPS) of G1-treated biofilms (control group -Figure 4A), G2-treated biofilms (EERP 3.25 mg - Figure 4B) and G3-treated biofilms (EERP 6.25 mg - Figure 4C).

The biofilm images obtained by confocal analysis are shown in Figure 4. In green, it is possible to observe the bacterial portion of biofilms while in red, the EPS portion. The third columns represent both portions with overlaid images. G3 reduced EPS production of biofilm without affecting bacterial portion when compared to the G1 treatment group.

### Kinetics of polymerization and degree of conversion (DC)

**Figure 5:**
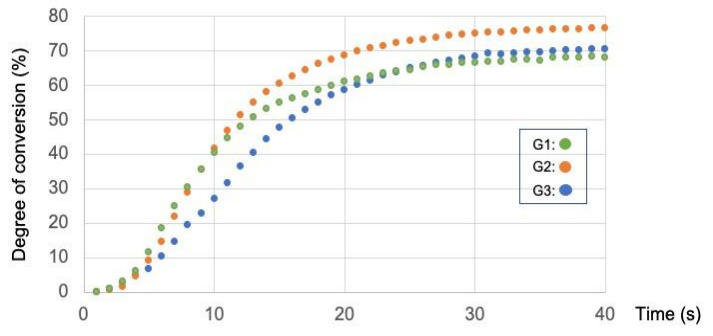
Polymerization kinetics graph during photoactivation for 40 seconds of the experimental orthodontic adhesives.

The polymerization kinetics of the orthodontic adhesives tested are depicted in Figure 5 and Table 1. G1 and G2 began the polymerization process before G3. The DC after 40 seconds of photoactivation ranged from 68.62% (± 1.22%) for G1, 70.75% (± 3.65%) for G3, to 76.85% (± 3.96%) for G2. The difference between G1 and G2 reached statistical significance (p < 0.05).

**Table 1:** Mean and standard deviation values of degree of conversion (DC) of the experimental orthodontic adhesives.

Groups	DC (%)
G1	68.62 (± 1.22)^B^
G2	76.85 (± 3.96)^A^
G3	70.75 (± 3.65)^B^

Different capital letters indicate a statistically significant difference (p<0.05).

### Bracket-Tooth Shear Bond Strength

The results of SBS tests are depicted in Table 2. One-way ANOVA revealed no statistically significant difference in SBS among the groups (p = 0.0984). The addition of EERP to the orthodontic primer did not affect the bracket-tooth SBS (p > 0.05), regardless of the concentration incorporated.

**Table 2:** Mean, Standard Deviation (SD), and 95% Confidence Interval (CI) of Shear Bond Strength values (in Megapascals) of the control and the experimental groups (n = 10).

	Mean	SD	95% CI Lower Bound	Upper Bound
G1 - Orthodontic primer (Control group)		6.6	15.5	25.0
G2 - Orthodontic primer + EERP 3.25 mg/mL	13.9 A*	6.1	8.8	19.0
G3 - Orthodontic primer + EERP 6.5 mg/mL	14.6 A*	7.0	8.7	20.5

* Similar capital letters indicate the absence of statistically significant differences (p > 0.05).

The frequencies and distribution of the ARI among groups are depicted in Table 3. The bond failure patterns were similar, when the different adhesives were used for bonding brackets. The most frequent failure pattern observed among the groups - ARI: 1, indicated that, in most cases, less than half of the enamel bonding site were covered with adhesive.

**Table 3:** Frequency and Distribution of Adhesive Remnant Index (ARI) of the control and the experimental groups.

	ARI Scores			
	0	1	2	3
G1 - Orthodontic primer (Control group)	3	5	2	0
G2 - Orthodontic primer + EERP 3.25 mg/mL	3	6	1	0
G3 - Orthodontic primer + EERP 6.5 mg/mL	3	5	2	0

* Fisher’s exact test was performed to analyze differences between groups at a significance level of α = 0.05.

## Discussion

Optimal oral hygiene combined with additional preventive measures might be extremely beneficial to avoid white spot lesion development; however, patients’ compliance is still challenging ^3^. In this scenario, bonding materials with antibacterial potential or bioactive properties have been developed ^21^. The advancement in this research field is indubitably promising since preventive methods that are less influenced by patients' motivation and collaboration seem to be more appropriate for the typical orthodontic patient ^22^.

The introduction of acid etching of dental enamel enabled the direct bonding of orthodontic brackets through composite materials ^23^. These materials are generally attached to the enamel surface after priming. Therefore, an adhesive with antimicrobial properties could potentially reduce biofilms and demineralization on the bracket/tooth interface ^6^. Yet, the definition of the optimal concentration of the antimicrobial agent to be added to the primer is critical since inadequate amounts might limit antimicrobial properties and excessive quantities might deteriorate the physic-chemical characteristics of the dental adhesives. The results from the present study showed that 6.5 mg/mL of EERP was the optimal concentration for antimicrobial activity, which also did not cause significant negative physic-chemical influence.

The polymerization kinetics indicated that for both controls and the 3.25 mg/mL-modified adhesive, the polymerization reaction started at the earliest time stage, as compared to the adhesive modified by EERP 6.5 mg/mL. The incorporation of 3.25 mg/mL of EERP into the orthodontic adhesive might have possibly decreased the viscosity of the material, increasing monomers chain mobility and the DC, as a consequence. Mechanical and biological composite properties tend to improve as the degree of conversion attained during photo-polymerization is increased ^24^. This may be due to the lower amount of uncured functional groups, which can act as plasticizers, reducing the mechanical properties and negatively interfering with the material biocompatibility ^25^. The DC achieved by all groups was in accordance with commercial adhesives ^26^. Therefore, although these parameters might have been, somehow, influenced by the presence of EERP, we suggest that this difference might not impact the clinical use of the modified adhesive. Additionally, SBS values observed for all groups, which indicate the mechanical resistance against bracket failure did not show statistically significant differences between groups.

Bracket-tooth SBS property has been widely studied and it is extremely important for orthodontic materials ^27^. Ideally, bracket-bonding strength should hold the attachment on the tooth surface throughout orthodontic treatment; and at the end of treatment, brackets should be detached without damaging the enamel ^28^. It has been estimated that orthodontic brackets require ideal bond strength values ranging from 5.9 MPa to 7.8 MPa; but many bonding systems exceed this requirement and achieve up to 11.1 MPa to 20.2 MPa, as in the case of Transbond XT composite resin ^29^ ^,^ ^30^. The results from the present study showed that all groups tested presented clinically acceptable bond strength values. Still, it is worth mentioning that, although no significant changes in bond failure patterns among the experimental and control groups have been detected, a higher data dispersion was observed for both experimental orthodontic adhesives tested. More studies testing different formulations should be performed aiming to achieve more predictable and consistent results.

Our results concerning the reduction of dry weight and the absence of significant differences in colony formation units are corroborated by the literature. A fraction obtained from Brazilian red propolis reduced the dry weight of *S. Mutans* biofilm, even though it did not alter colony formation units through a very similar model as the one we used here ^15^. In fact, the same article ^15^ demonstrated this fraction from Brazilian red propolis acts by reducing the exopolysaccharide produced by *S. Mutans*. Therefore, since the present article showed no statistical difference in the formation of bacteria colonies, seems that the primer modified by EERP acts through the same mechanisms, by inhibiting EPS production and without affecting the bacterial amount of *S. Mutans* biofilm. To verify this hypothesis, we performed CLMS analysis to evaluate the EPS production by *S. Mutans* biofilm. Indeed, both concentrations of EERP-modified primer significantly reduced the EPS content of biofilms (Figure 3).

*S. Mutans* utilizes host-provided sucrose to modulate the development of cariogenic biofilms, producing EPSs and acid. This bacterial species is highly acidogenic and aciduric (survives in acidic environments). The EPS offers attaching sites for bacterial colonization and local accumulation of *S. Mutans* and other microorganisms on the tooth's surface. As time passes, EPS accumulation becomes a highly consistent and diffusion-restrictive matrix, protecting the embedded bacteria and promoting acid accumulation and low pH values locally ^15^. Therefore, EPS production is a key target of *S. Mutans* virulence factor.

The dental orthodontic EERP-modified primer impaired the virulence of biofilm since *S. Mutans* cells without EPS-building properties are compromised in their capacity to produce and sustain acidic microenvironments. Thus, this novel material could be a promising agent against EPS-matrix development, which could mitigate both the increase and virulence of cariogenic *S. Mutans* biofilms formed in the oral cavity of orthodontic patients. The present study is the first to incorporate Brazilian red propolis into an orthodontic material, demonstrating the potential of natural antimicrobial agents to be incorporated into dental materials and improve the antimicrobial properties thus reducing clinical lesions such as dental caries due to orthodontic treatments. Clinically, this finding may represent a decreased biofilm formation around brackets during the initial phase of orthodontic treatment. As a result, the incidence of white spot lesions around brackets may be reduced. However, clinical studies should be performed to determine whether the *in vitro* findings would prevail *in vivo*.

Considering the limitations of *in vitro* evaluations in simulating modifications of sugar exposure, pH changes, and biofilm formation of the oral environment, *in situ* models and clinical studies should be still performed, to verify the stability and longevity of this antimicrobial effect throughout the fixed orthodontic treatment. In this way, the present manuscript should be interpreted as an initial study demonstrating that the addition of propolis to orthodontic dental materials has the potential to improve biofilm control.

## Conclusion

It was concluded that the addition of 6.5mg/mL of Brazilian red propolis to orthodontic adhesive showed significant antimicrobial activity and did not significantly alter its physicochemical properties tested.

## Data Availability

The datasets used and/or analyzed during the current study are available from the corresponding author upon reasonable request.

## References

[1] Verrusio C, Iorio-Siciliano V, Blasi A, Leuci S, Adamo D, Nicolò M (2018). The effect of orthodontic treatment on periodontal tissue
inflammation: A systematic review. Quintessence Int.

[2] Tanner AC, Sonis AL, Lif Holgerson P, Starr JR, Nunez Y, Kressirer CA, Paster BJ, Johansson I (2012). White-spot lesions and gingivitis microbiotas in orthodontic
patients. J Dent Res.

[3] Julien KC, Buschang PH, Campbell PM (2013). Prevalence of white spot lesion formation during orthodontic
treatment. Angle Orthod.

[4] Guzmán-Armstrong S, Chalmers J, Warren JJ (2010). Ask us. White spot lesions: prevention and
treatment. Am J Orthod Dentofacial Orthop.

[5] Olivieri A, Ferro R, Benacchio L, Besostri A, Stellini E (2013). Validity of Italian version of the Child Perceptions
Questionnaire (CPQ11-14). BMC Oral Health.

[6] Chung SH, Cho S, Kim K, Lim BS, Ahn SJ (2017). Antimicrobial and physical characteristics of orthodontic primers
containing antimicrobial agents. Angle Orthod.

[7] Islam A, Da Silva JG, Berbet FM, da Silva SM, Rodrigues BL, Beraldo H, Melo MN, Frézard F, Demicheli C (2014). Novel triphenylantimony(V) and triphenylbismuth(V) complexes with
benzoic acid derivatives: structural characterization, in vitro
antileishmanial and antibacterial activities and cytotoxicity against
macrophages. Molecules.

[8] Geraldeli S, Maia Carvalho LA, de Souza Araújo IJ, Guarda MB, Nascimento MM, Bertolo MVL, Di Nizo PT, Sinhoreti MAC, McCarlie VW (2021). Incorporation of Arginine to Commercial Orthodontic Light-Cured
Resin Cements-Physical, Adhesive, and Antibacterial
Properties. Materials.

[9] André CB, Rosalen PL, Giannini M, Bueno-Silva B, Pfeifer CS, Ferracane JL (2021). Incorporation of Apigenin and tt-Farnesol into dental composites
to modulate the Streptococcus mutans virulence. Dent Mater.

[10] Pereira ML, Santos DCP, Soares CAM, Bazan TAXN, Bezerra CM, Silva MVD, Correia MTDS, Cardenas AFM, Siqueira FSF, Carvalho EM, Fronza BM, André CB, Nascimento da Silva LC, Galvão LCC (2022). Development and Physicochemical Characterization of Eugenia
brejoensis Essential Oil-Doped Dental Adhesives with Antimicrobial Action
towards Streptococcus mutans. J Funct Biomater.

[11] Inagaki LT, Dainezi VB, Alonso RC, Paula AB, Garcia-Godoy F, Puppin-Rontani RM, Pascon FM (2016). Evaluation of sorption/solubility, softening, flexural strength
and elastic modulus of experimental resin blends with
chlorhexidine. J Dent.

[12] Melo MAS, Morais WA, Passos VF, Lima JPM, Rodrigues LKA (2014). Fluoride releasing and enamel demineralization around orthodontic
brackets by fluoride-releasing composite containing
nanoparticles. Clin Oral Investig.

[13] Bueno-Silva B, Marsola A, Ikegaki M, Alencar SM, Rosalen PL (2017). The effect of seasons on Brazilian red propolis and its botanical
source: chemical composition and antibacterial activity. Nat Prod Res.

[14] Bueno-Silva B, Alencar SM, Koo H, Ikegaki M, Silva GV, Napimoga MH, Rosalen PL (2013). Anti-inflammatory and antimicrobial evaluation of neovestitol and
vestitol isolated from Brazilian red propolis. J Agric Food Chem.

[15] Bueno-Silva B, Koo H, Falsetta ML, Alencar SM, Ikegaki M, Rosalen PL (2013). Effect of neovestitol-vestitol containing Brazilian red propolis
on accumulation of biofilm in vitro and development of dental caries in
vivo. Biofouling.

[16] Miranda SLF, Damasceno JT, Faveri M, Figueiredo L, da Silva HD, Alencar SMA, Rosalen PL, Feres M, Bueno-Silva B (2019). Brazilian red propolis reduces orange-complex periodontopathogens
growing in multispecies biofilms. Biofouling.

[17] de Figueiredo KA, da Silva HDP, Miranda SLF, Gonçalves FJDS, de Sousa AP, de Figueiredo LC, Feres M, Bueno-Silva B (2020). Brazilian Red Propolis Is as Effective as Amoxicillin in
Controlling Red-Complex of Multispecies Subgingival Mature Biofilm In
Vitro. Antibiotics (Basel).

[18] Bim-Júnior O, Gaglieri C, Bedran-Russo AK, Bueno-Silva B, Bannach G, Frem R, Ximenes VF, Lisboa-Filho PN (2020). MOF-Based Erodible System for On-Demand Release of Bioactive
Flavonoid at the Polymer-Tissue Interface. ACS Biomater Sci Eng.

[19] Freires IA, Bueno-Silva B, Galvão LC, Duarte MC, Sartoratto A, Figueira GM, de Alencar SM, Rosalen PL (2015). The Effect of Essential Oils and Bioactive Fractions on
Streptococcus mutans and Candida albicans Biofilms: A Confocal
Analysis. Evid Based Complement Alternat Med.

[20] Ely C, Schneider LF, Ogliari FA, Schmitt CC, Corrêa IC, Lima Gda S, Samuel SM, Piva E (2012). Polymerization kinetics and reactivity of alternative initiators
systems for use in light-activated dental resins. Dent Mater.

[21] Nascimento PLMM, Meereis CTW, Maske TT, Ogliari FA, Cenci MS, Pfeifer CS, Faria-E-Silva AL (2017). Addition of ammonium-based methacrylates to an experimental
dental adhesive for bonding metal brackets: Carious lesion development and
bond strength after cariogenic challenge. Am J Orthod Dentofacial Orthop.

[22] van der Veen MH, Mattousch T, Boersma JG (2007). Longitudinal development of caries lesions after orthodontic
treatment evaluated by quantitative light-induced
fluorescence. Am J Orthod Dentofacial Orthop.

[23] Mandall NA, Hickman J, Macfarlane TV, Mattick RC, Millett DT, Worthington HV (2018). Adhesives for fixed orthodontic brackets. Cochrane Database Syst Rev.

[24] Lovell LG, Lu H, Elliott JE, Stansbury JW, Bowman CN (2001). The effect of cure rate on the mechanical properties of dental
resins. Dent Mater.

[25] Yap AU, Soh MS, Han TT, Siow KS (2004). Influence of curing lights and modes on cross-link density of
dental composites. Oper Dent.

[26] Corekci B, Malkoc S, Ozturk B, Gunduz B, Toy E (2011). Polymerization capacity of orthodontic composites analyzed by
Fourier transform infrared spectroscopy. Am J Orthod Dentofacial Orthop.

[27] Finnema KJ, Ozcan M, Post WJ, Ren Y, Dijkstra PU (2010). In-vitro orthodontic bond strength testing: a systematic review
and meta-analysis. Am J Orthod Dentofacial Orthop.

[28] Chalipa J, Jalali YF, Gorjizadeh F, Baghaeian P, Hoseini MH, Mortezai O (2016). Comparison of Bond Strength of Metal and Ceramic Brackets Bonded
with Conventional and High-Power LED Light Curing Units. J Dent.

[29] Rajagopal R, Padmanabhan S, Gnanamani J (2004). A comparison of shear bond strength and debonding characteristics
of conventional, moisture-insensitive, and self-etching primers in
vitro. Angle Orthod.

[30] Rix D, Foley TF, Mamandras A (2001). Comparison of bond strength of three adhesives: composite resin,
hybrid GIC, and glass-filled GIC. Am J Orthod Dentofacial Orthop.

